# Beyond Tradition: Non-surgical Endodontics and Vital Pulp Therapy as a Dynamic Combination

**DOI:** 10.7759/cureus.44134

**Published:** 2023-08-25

**Authors:** Neha K Urkande, Nikhil Mankar, Pradnya P Nikhade, Manoj Chandak

**Affiliations:** 1 Conservative Dentistry and Endodontics, Sharad Pawar Dental College And Hospital, Datta Meghe Institute of Higher Education & Research, Wardha, IND

**Keywords:** mature permanent molars, combination approach, vital pulp therapy, non-surgical endodontic treatment, apical periodontitis, symptomatic irreversible pulpitis

## Abstract

Symptomatic irreversible pulpitis and apical periodontitis in mature permanent teeth present challenges in their management. Traditional treatment approaches, such as root canal therapy or tooth extraction, may compromise tooth structure and oral function. This review article explores the combination of non-surgical endodontic treatment and vital pulp therapy as an alternative approach for these conditions. The purpose is to examine this combined approach's effectiveness, benefits, challenges, and limitations. The objectives include reviewing the literature, evaluating clinical outcomes, discussing potential benefits, and providing recommendations for clinical practice. The combination approach aims to preserve tooth structure, promote healing, and reduce postoperative complications. The article discusses the rationale for combining the two techniques, presents evidence supporting their efficacy, and outlines the techniques and protocols involved. Clinical outcomes, case studies, potential challenges, and comparative analysis with traditional approaches are also explored. Future directions and research recommendations highlight areas for further investigation, innovations, and the development of clinical guidelines. In conclusion, the combination of non-surgical endodontic treatment and vital pulp therapy offers a valuable strategy for managing mature permanent mandibular molars with symptomatic irreversible pulpitis and apical periodontitis. Further research and advancements are needed to refine the treatment protocol and expand the evidence base, and clinicians should stay updated to provide optimal care and improve patient outcomes.

## Introduction and background

Symptomatic irreversible pulpitis and apical periodontitis present substantial difficulties in managing mature permanent teeth. These conditions are marked by intense dental pain and inflammation, causing significant discomfort and impairment in daily activities for affected individuals. Prompt intervention is often necessary to alleviate the symptoms and prevent the progression of these conditions into more severe forms, such as periapical abscesses or tooth loss [1,2]. Traditionally, root canal therapy or tooth extraction has been the primary treatment for symptomatic irreversible pulpitis and apical periodontitis. Root canal therapy involves removing the infected pulp tissue from the tooth's root canals and sealing them to prevent further infection. On the other hand, tooth extraction involves removing the entire affected tooth. While these methods can effectively address the infection and alleviate symptoms, they have certain drawbacks [3,4].

One significant drawback is the potential loss of the natural tooth structure, which can affect aesthetics and function. Losing a tooth can impact a person's ability to chew, speak, and smile confidently. Moreover, extracting a permanent tooth often necessitates additional dental procedures, such as dental implants or bridges, to restore oral function and aesthetics. These procedures can be invasive, time-consuming, and costly, increasing patients' burden [5,6]. Given these limitations, there is a growing need to explore alternative strategies that can effectively manage symptomatic irreversible pulpitis and apical periodontitis while preserving the integrity of the natural tooth. This has led to the emergence of the combination approach, involving integrating non-surgical endodontic treatment and vital pulp therapy. This approach aims to maintain the vitality of the tooth and promote healing while addressing the infection and inflammation associated with these conditions [7,8].

By combining non-surgical endodontic treatment, which involves thorough disinfection and shaping of the root canals, with vital pulp therapy, which focuses on preserving and promoting the health of the remaining vital pulp tissue, clinicians can achieve favorable treatment outcomes. This approach seeks to balance eradicating the infection and preserving the tooth's structure and function, ultimately enhancing the long-term prognosis for patients with symptomatic irreversible pulpitis and apical periodontitis [9,10]. Exploring and understanding these alternative strategies is crucial as they offer potential benefits over traditional treatment approaches. By preserving the natural tooth, patients can retain a functional dentition, maintaining proper occlusion and preventing the need for additional restorative procedures. Furthermore, preserving the vitality of the remaining pulp tissue may contribute to enhanced healing, dentin regeneration, and immune response, further promoting long-term success and reducing the risk of future complications [11,12].

This review article explores the combination of non-surgical endodontic treatment and vital pulp therapy for managing mature permanent mandibular molars with symptomatic irreversible pulpitis and apical periodontitis. By consolidating existing evidence and evaluating clinical outcomes, this review provides insights into the effectiveness and benefits of this combined approach. The objectives include a comprehensive literature review, evaluation of success rates and clinical outcomes, discussion of potential benefits, and providing recommendations for clinical practice and future research. This review article contributes to the existing literature, assists clinicians in evidence-based decision-making, and highlights the potential of this combined approach in preserving natural teeth and improving patient outcomes.

## Review

Factors contributing to symptomatic irreversible pulpitis and apical periodontitis

Symptomatic irreversible pulpitis and apical periodontitis result from complex interactions between various factors. Dental caries, which demineralizes tooth structures by acid-producing bacteria, is a primary cause of pulp inflammation. Inviting bacteria into the pulp chamber can lead to infection and subsequent inflammation. Traumatic dental injuries, such as fractures or cracks in the tooth, can also expose the pulp to bacteria and irritants, triggering an inflammatory response. Other factors contributing to these conditions include deep restorations, excessive tooth wear, and iatrogenic factors related to dental procedures [13,14].

The inflammation and infection of the dental pulp can lead to symptomatic irreversible pulpitis, characterized by severe and persistent dental pain that is unresponsive to stimuli and often accompanied by swelling and sensitivity. If left untreated, the inflammatory process can extend to the tissues surrounding the tooth's root, resulting in apical periodontitis. Apical periodontitis is characterized by inflammation and destruction of the periapical tissues, including the periodontal ligament and alveolar bone. The presence of apical periodontitis is often diagnosed radiographically by the presence of periapical radiolucency [15,16].

Understanding the anatomy and pathophysiology of mature permanent mandibular molars is crucial for comprehending the underlying mechanisms of symptomatic irreversible pulpitis and apical periodontitis. By identifying the factors contributing to these conditions, clinicians can develop appropriate treatment strategies to manage the symptoms, eliminate infection, and preserve the integrity of the tooth and surrounding structures [17].

Traditional treatment approaches

Root Canal Therapy

Root canal therapy, or endodontic therapy, is a dental procedure aimed at treating and preserving a tooth that has suffered from extensive decay, infection, or trauma affecting its innermost structures, namely the pulp chamber and root canals. The procedure involves the removal of infected or inflamed pulp tissue, which comprises nerves, blood vessels, and connective tissue, from within the tooth's pulp chamber and root canals. Subsequently, the void left by the removed pulp is meticulously cleaned, disinfected, shaped, and then filled with a biocompatible material to prevent recontamination. Root canal therapy aims to eliminate pain, halt the spread of infection, and salvage the affected tooth, thereby preserving its function and structure [4].

Clinical considerations and limitations: Root canal therapy has a high success rate and is considered the gold standard treatment for these conditions. However, there are several clinical considerations and limitations to be aware of. The complexity of the root canal system in mandibular molars poses challenges in achieving thorough cleaning and complete obturation of all canals. Factors such as calcifications, curved canals, and accessory canals can make the procedure technically demanding and increase the risk of procedural errors. Additionally, there is a potential for postoperative complications, such as instrument fracture, root perforation, and postoperative pain [18-20].

Extraction

Extraction, in dentistry, refers to the deliberate and controlled removal of a tooth from its designated socket in the jawbone. This procedure becomes necessary when a tooth is adversely affected by conditions such as severe decay, infection, structural damage, periodontal disease, or when it poses a risk to overall oral health. The extraction process involves a sequence of steps, starting with administering local anesthesia to ensure patient comfort. Subsequently, specialized instruments gently loosen the tooth from the surrounding bone and soft tissues. Depending on the case's complexity, the tooth may be fully erupted or impacted, a condition where the tooth fails to emerge from the gum line. In the case of impacted teeth, a surgical extraction technique may be required, involving the creation of a small incision and the removal of bone to access the tooth [20].

Non-surgical endodontic treatment

Standard non-surgical endodontic treatment for mature permanent mandibular molars with symptomatic irreversible pulpitis and apical periodontitis involves the removal of infected or inflamed pulp tissue from the tooth's root canals, followed by cleaning, shaping, and filling the canals to prevent reinfection. This procedure aims to eliminate the source of infection, alleviate pain, and promote healing [18].

Vital pulp therapy

Pulpotomy

The pulpotomy partially removes the inflamed or infected pulp tissue, specifically targeting the coronal portion. The remaining healthy pulp tissue is medicated, and biocompatible material, such as calcium hydroxide or mineral trioxide aggregate (MTA), is placed to promote healing and protect the remaining pulp tissue. This approach aims to maintain the vitality of the remaining pulp and allow for continued root development in immature teeth [21,22].

Direct pulp capping: Direct pulp capping involves the application of a medicament or biocompatible material directly on an exposed or nearly exposed pulp, typically resulting from caries excavation or trauma. The material forms a seal to protect the pulp and stimulate dentin bridge formation, allowing for potential pulp healing and continued vitality [23].

Indirect pulp capping: Indirect pulp capping is performed when the pulp is not directly exposed but is at risk of inflammation due to deep caries. A layer of protective material, such as calcium hydroxide or resin-based materials, is placed over the deepest carious dentin to promote pulp vitality and prevent the further progression of caries. This approach aims to allow the pulp to recover and minimize the need for invasive procedures [24].

Challenges and Outcomes of Vital Pulp Therapy

Vital pulp therapy techniques, including pulpotomy, direct pulp capping, and indirect pulp capping, have been devised to uphold pulp vitality and expedite healing. While these techniques have succeeded in select scenarios, their suitability is predominantly observed in immature teeth endowed with vital pulp tissue [25,26]. Moreover, the scope of vital pulp therapy is relatively limited when applied to mature teeth characterized by substantial inflammation and infection. In such cases, these techniques may offer suboptimal outcomes due to the compromised condition of the pulp. Furthermore, it's crucial to recognize that these approaches might not directly address the root cause of pulpitis and apical periodontitis, necessitating supplementary treatments or interventions for comprehensive resolution.

Combination of non-surgical endodontic and vital pulp therapy

The Rationale for Combining the Two Techniques

The combination of non-surgical endodontic treatment and vital pulp therapy for managing mature permanent mandibular molars with symptomatic irreversible pulpitis and apical periodontitis is based on a sound rationale. Non-surgical endodontic treatment eliminates infection from the root canals, while vital pulp therapy aims to preserve the remaining healthy pulp tissue. By combining these two techniques, clinicians can address both the source of infection and inflammation while maintaining the vitality of the tooth, leading to improved treatment outcomes and potential long-term success [26-28].

Evidence Supporting the Combination Approach

Studies have investigated the outcomes of combining non-surgical endodontic treatment and vital pulp therapy and reported favourable results, demonstrating successful resolution of symptoms, healing of periapical tissues, and preservation of tooth vitality. They have highlighted the potential of this combined approach in managing symptomatic irreversible pulpitis and apical periodontitis while maintaining the tooth's structural integrity [29].

Long-term Success Rates

Long-term follow-up studies have shown promising success rates for the combination approach. These have reported high survival rates, the absence of persistent or recurrent symptoms, and maintenance of tooth functionality. The preservation of tooth structure and the prevention of tooth loss have been demonstrated as significant advantages of this approach compared to traditional treatment methods [30,31].

Advantages and potential benefits

Preservation of Tooth Structure and Function

One of the key advantages of the combination approach is preserving the natural tooth structure. Patients can maintain proper occlusion, chewing efficiency, and aesthetics by retaining the tooth. Preserving the tooth eliminates additional restorative procedures, such as dental implants or bridges, which may have limitations and complications [32,33].

Enhanced Healing and Regeneration Potential

Combining non-surgical endodontic treatment and vital pulp therapy can enhance healing and regeneration. By preserving the essential pulp tissue, which contains stem cells and growth factors, the regenerative capacity of the tooth may be promoted. This can lead to new dentin formation, improved periapical healing, and reduced likelihood of future complications [34].

Reduced Postoperative Complications

Compared to traditional treatment approaches, the combination approach has been associated with reduced postoperative complications. Preserving vital pulp tissue can minimize pain and inflammation. Avoiding more invasive procedures, such as tooth extraction, can also decrease the risk of surgical complications and accelerate recovery [35].

Techniques and protocols for the combination approach

Proper case selection and accurate diagnosis are essential for successfully implementing the combination approach. Patients with mature permanent mandibular molars exhibiting symptomatic irreversible pulpitis and apical periodontitis should be considered for this treatment. Diagnostic criteria may include clinical symptoms such as severe dental pain, sensitivity, swelling, and radiographic evidence of periapical radiolucency. Careful assessment of the patient's oral health, medical history, and the extent of pulp and periapical involvement is crucial for selecting appropriate cases [8,12,14,16,19].

Step-by-step procedure for non-surgical endodontic and vital pulp therapy combination

Local Anesthesia and Isolation Methods

The procedure begins with the administration of local anaesthesia to ensure patient comfort. Rubber dam isolation is then employed to maintain a clean and dry operating field, preventing contamination and improving visibility during the treatment [36]. Local anaesthetics, such as lidocaine or articaine, are commonly used for achieving profound anaesthesia. Rubber dam isolation, appropriate clamps, and dental floss isolate the tooth and create a sterile operating field [37,38].

Instrumentation and Disinfection Protocols

Carefully removing the carious or damaged tooth structure gives access to the pulp chamber. The root canal system is thoroughly explored and cleaned using appropriate endodontic instruments, such as files and irrigating solutions. Mechanical debridement and irrigation protocols are crucial for completely removing infected or inflamed pulp tissue and disinfection of the root canals [39]. Endodontic hand files and rotary instruments are utilized for cleaning and shaping the root canals. Irrigating solutions, such as sodium hypochlorite or chlorhexidine, are used for disinfection. Obturation materials, such as gutta-percha and sealer, fill root canals [40,41].

Selection and Application of Vital Pulp Therapy Materials

After cleaning and disinfecting the root canals, vital pulp therapy materials are selected and applied according to the chosen technique. For pulpotomy, the coronal portion of the pulp is partially removed, and biocompatible material, such as calcium hydroxide or MTA, is placed to promote healing and protect the remaining vital pulp tissue. In direct pulp capping, a medicament or biocompatible material is applied directly on the exposed or nearly exposed pulp to stimulate dentin bridge formation and pulp healing. Indirect pulp capping involves placing a protective material over deep carious dentin to promote pulp vitality and prevent further progression of caries [9,42].

The selection of vital pulp therapy materials depends on the specific technique employed. Calcium hydroxide and MTA are commonly used for pulpotomy procedures. Direct pulp capping materials may include calcium hydroxide, resin-based materials, or bioactive dentin substitutes. For indirect pulp capping, calcium hydroxide or resin-based materials are frequently utilized. It is important to note that the specific techniques, materials, and protocols may vary depending on the clinician's preference, the patient's condition, and available resources. Adherence to proper infection control measures and following evidence-based guidelines are crucial to ensure optimal outcomes and minimize the risk of complications [22,43].

Evaluation of treatment success

Pain Relief and Resolution of Symptoms

The evaluation of treatment success in the combination approach for managing mature permanent mandibular molars with symptomatic irreversible pulpitis and apical periodontitis includes the assessment of pain relief and resolution of symptoms. Patients should experience a significant reduction or complete elimination of dental pain and associated symptoms following treatment. Subjective reports from patients and the use of pain scales can help evaluate the level of pain relief achieved [44].

Radiographic Assessment of Periapical Healing

Radiographic evaluation is essential for assessing periapical healing following the combination approach. Pre- and post-treatment radiographs are compared to determine the resolution or reduction in periapical radiolucency, indicating healing of the apical periodontitis. The absence of periapical radiolucency or a decrease in size indicates successful periapical recovery [45].

Patient Demographics, Presenting Symptoms, and Diagnosis

Case studies presenting successful outcomes of the combination approach should include detailed information about the patient's demographics, including age and gender. The presenting symptoms and the diagnosis of symptomatic irreversible pulpitis and apical periodontitis should also be described. This includes information on the severity of pain, sensitivity, swelling, and radiographic findings [46].

Treatment Procedure and Follow-up Evaluation

The case studies should detail the treatment procedure, including the techniques and materials used in the combination approach. The step-by-step process should be explained, from local anaesthesia and isolation to non-surgical endodontic treatment and vital pulp therapy. Any modifications or considerations specific to the case should be highlighted. A case follow-up evaluation should also be included to assess treatment outcomes. This may involve multiple post-treatment visits over a specified period. The assessment should include information on the patient's postoperative symptoms, healing progression, and radiographic findings. Follow-up radiographs and additional tests, such as vitality testing or cone-beam computed tomography (CBCT), should be included to provide objective evidence of treatment success [47].

By presenting case studies illustrating successful outcomes, clinicians can showcase the efficacy and benefits of the combination approach in managing mature permanent mandibular molars with symptomatic irreversible pulpitis and apical periodontitis. These case studies serve as valuable evidence supporting the implementation of this treatment modality and can guide clinicians in making informed decisions for optimal patient care [48].

Potential challenges and limitations

Treatment Complexities and Potential Complications

The combination of non-surgical endodontic treatment and vital pulp therapy for managing mature permanent mandibular molars with symptomatic irreversible pulpitis and apical periodontitis may present certain complexities and potential complications. These can include challenges associated with accessing and adequately cleaning complex root canal systems, ensuring complete disinfection of the pulp space, and achieving proper sealing of the root canals. Furthermore, the potential for procedural errors such as instrument fracture or perforation should be considered. Clinicians must be proficient in endodontic techniques and possess the necessary skills to address these complexities and prevent complications [49].

Factors Influencing Treatment Success

Several factors can influence the success of the combination approach. Pre-existing conditions such as extensive caries, root resorption, or previous endodontic treatments may affect treatment outcomes. Additionally, the patient's overall oral health, systemic health, and compliance with post-treatment care instructions can impact the success of the treatment. Adequate patient selection, accurate diagnosis, and appropriate case management are crucial to favourable treatment outcomes [50].

Comparative analysis with traditional treatment approaches

A comparative analysis between the combination approach and traditional treatment approaches is essential to understand the advantages and limitations of each method. While conventional techniques such as root canal therapy or tooth extraction have been widely utilized, they may result in the loss of natural tooth structure and compromised oral function. The combination approach, on the other hand, aims to preserve tooth structure, maintain vitality, and promote healing and regeneration. Comparative studies evaluating the success rates, clinical outcomes, and long-term prognosis of the combination approach versus traditional approaches can provide valuable insights for clinicians in selecting the most appropriate treatment modality [51].

By addressing the potential challenges and limitations associated with the combination approach, clinicians can be better prepared to manage and mitigate any complications that may arise. Understanding the factors influencing treatment success and conducting comparative analyses with traditional treatment approaches can aid in evidence-based decision-making and improve patient outcomes in managing mature permanent mandibular molars with symptomatic irreversible pulpitis and apical periodontitis [52].

Future directions and research recommendations

Areas for Further Investigation

More studies with extended follow-up periods are needed to evaluate the combination approach's long-term success rates, durability, and stability. Comparative studies directly comparing the combination approach with traditional treatment approaches, such as root canal therapy or tooth extraction, are necessary to establish the superiority or non-inferiority of the combination approach in terms of clinical outcomes, patient satisfaction, and cost-effectiveness. Assessing patient-reported outcomes, such as quality of life, functional outcomes, and patient satisfaction, will provide valuable insights into the impact of the combination approach on patients' overall well-being. Exploring regenerative techniques, including stem cell-based therapies and tissue engineering approaches, can further enhance the healing and regeneration potential of the combination approach. The field of dentistry is constantly evolving, and several innovations and emerging technologies can influence the future direction of the combination approach. These may include continued research and development of new biocompatible materials such as bioceramics or bioactive scaffolds, which can improve the outcomes of vital pulp therapy and promote pulp healing and regeneration. Advanced imaging techniques, such as cone-beam computed tomography (CBCT) or optical coherence tomography (OCT), can enhance the diagnosis, treatment planning, and evaluation of treatment outcomes in the combination approach. The development of minimally invasive techniques, such as laser-assisted endodontics or ultrasonic irrigation, can reduce treatment complexities, enhance disinfection, and improve patient comfort.

Clinical Guidelines and Consensus Statements

Establishing clinical guidelines and consensus statements can provide standardized protocols and recommendations for implementing the combination approach. Expert panels and professional organizations should collaborate to develop evidence-based guidelines that address patient selection criteria, treatment protocols, materials, and follow-up procedures. By focusing on these future directions and research recommendations, the field can further advance the understanding and application of the combination approach for managing mature permanent mandibular molars with symptomatic irreversible pulpitis and apical periodontitis. Continued research and innovation will improve treatment outcomes, expand treatment options, and ultimately enhance patient care in these challenging cases.

## Conclusions

Combining non-surgical endodontic treatment with vital pulp therapy is valuable for managing mature permanent mandibular molars with symptomatic irreversible pulpitis and apical periodontitis. This approach preserves natural tooth structure, maintains functionality, and offers advantages over traditional methods like root canal therapy or extraction. It promotes healing and regeneration, enhancing long-term outcomes and preserving tooth vitality. However, further research and advancements are needed to refine the treatment protocol and establish it as a standard modality based on robust evidence. Ongoing scientific investigation and clinical studies will provide valuable data for evidence-based decision-making in practice.
